# Transcriptional responses to *Fusarium oxysporum* f. sp. *lycopersici* (Sacc.) Snyder & Hansen infection in three Colombian tomato cultivars

**DOI:** 10.1186/s12870-021-03187-z

**Published:** 2021-09-08

**Authors:** Walter Ricardo López, Dora Janeth Garcia-Jaramillo, Nelson Ceballos-Aguirre, Jairo Castaño-Zapata, Ricardo Acuña-Zornosa, Juan Jovel

**Affiliations:** 1 Departamento de Física y Química. Facultad de Ciencias Naturales, Universidad Nacional de Colombia sede Manizales, Manizales, Caldas, Colombia; 2 Graduate School of Agricultural Sciences. Facultad de Ciencias Agropecuarias, Universidad de Caldas, Manizales, Caldas, Colombia; 3 Departamento de Producción Agropecuaria. Facultad de Ciencias Agropecuarias, Universidad de Caldas. Manizales, Caldas, Colombia; 4 Research Office. Faculty of Medicine and Dentistry, University of Alberta, Edmonton, Alberta Canada

**Keywords:** Tomato, *Fusarium oxysporum*, RNAseq, Resistance, Selection of cultivars

## Abstract

**Background:**

*Fusarium oxysporum* f. sp. *lycopersici* (*Fol)* is a compendium of pathogenic and non-pathogenic fungal strains. Pathogenic strains may cause vascular wilt disease and produce considerable losses in commercial tomato plots. To gain insight into the molecular mechanisms mediating resistance to *Fol* in tomato, the aim of our study was to characterize the transcriptional response of three cultivars (CT1, CT2 and IAC391) to a pathogenic (*Fol*-pt) and a non-pathogenic *(Fo*-npt*)* strain of *Fo*.

**Results:**

All cultivars exhibited differentially expressed genes in response to each strain of the fungus at 36 h post-inoculation. For the pathogenic strain, CT1 deployed an apparent active defense response that included upregulation of WRKY transcription factors, an extracellular chitinase, and terpenoid-related genes, among others. In IAC391, differentially expressed genes included upregulated but mostly downregulated genes. Upregulated genes mapped to ethylene regulation, pathogenesis regulation and transcription regulation, while downregulated genes potentially impacted defense responses, lipid transport and metal ion binding. Finally, CT2 exhibited mostly downregulated genes upon *Fol*-pt infection. This included genes involved in transcription regulation, defense responses, and metal ion binding.

**Conclusions:**

Results suggest that CT1 mounts a defense response against *Fol*-pt. IAC391 exhibits an intermediate phenotype whereby some defense response genes are activated, and others are suppressed. Finally, the transcriptional profile in the CT2 hints towards lower levels of resistance. *Fo*-npt also induced transcriptional changes in all cultivars, but to a lesser extent. Results of this study will support genetic breeding programs currently underway in the zone.

**Supplementary Information:**

The online version contains supplementary material available at 10.1186/s12870-021-03187-z.

## Background

The plant immune system responds to pathogen attack by deploying two main defense strategies. i) recognition of pathogen-associated molecular patterns (PAMPs), such as chitin and β-glucans, by transmembrane pattern recognition receptors (PRRs), and ii) mounting a poorly-understood intracellular molecular defense response upon detection of plant proteins that have been activated by pathogen effectors, using polymorphic NB-LRR proteins [1]. Defense responses mediated by NB-LRR proteins are effective against biotrophic and hemibiotrophic pathogens, such as *Fusarium oxysporum* [2]. In the zig-zag model of plant-pathogen coevolution, plant PRRs detect PAMPs and activate pathogen-triggered immunity (PTI). Pathogens antagonize PTI leading to effector-triggered susceptibility (ETS). Intracellularly, NB-LRRs recognize pathogen effectors and restrict infection, triggering an amplified form of PTI, often associated with a hypersensitive response, dubbed effector-triggered immunity (ETI). Pathogens harboring effector mutants that are not recognized by NB-LRRs escape or suppress ETI. In turn, selection favors new host cells with polymorphic NB-LRRs that do recognize mutant effectors reinstating ETI [3]. As a result, antagonistic molecular encounters between the pathogen and the plant cell ignite a cascade of transcriptional and post-transcriptional events that either result in disease or resistance and can spread systemically through the plant [4, 5]. Understanding such transcriptional responses is of paramount importance for the comprehension of disease dynamics and for the design of management strategies.

*Fusarium oxysporum* constitutes an ensemble of strains that cause vascular wilt diseases in many cash crops worldwide and was initially described and taxonomically classified by Snyder & Hansen in 1940 [6, 7] . Although sexual reproduction has not been documented for all species, it is thought that lateral gene transfer may be responsible for the genetic and pathogenic diversity observed in the *Fusarium* complex [8]. *Fusarium oxysporum* f. sp. *lycopersici* (*Fol*) is a severe pathogen of tomato. It comprises three races with variable virulence and causes losses between 21 and 47% [9] in tropical and subtropical regions of the world [10]. *F. oxysporum* is considered a hemibiotrophic fungus that systemically invades the plant vascular system and eventually kills its hosts [2]. *Fol* secretes mycotoxins that activate defense responses in the plant, including callose deposition, activation of the jasmonic acid pathway and proliferation of parenchymatic cells [11, 12]. Non-pathogenic strains of *Fo* have also been reported [6], which although incapable of causing vascular wilt, colonize roots and can indeed act as biological controllers of pathogenic strains because they compete for nutrients and activate plant defense responses [13–16].

In wild tomato (*Solanum pennelli*), resistance genes against *Fol* are found and they are called *I* (for immunity), *I-2* and *I-3,* which have been introgressed into a number of commercial cultivars [17, 18]. In turn, *Fol* encodes avirulence (avr) genes that are recognized by products of *I* genes. Some mutations in avr genes overcome *I*-mediated resistance giving rise to *Fol* pathogenic races. This poses an arm race between tomato resistance genes and avr *Fol* genes [19]. To manage pathogenic races of *Fol* against which *I* genes fail to confer resistance, a variety of strategies have been assayed. For instance, salicylic acid has been used to prime systemic acquired resistance, which leads to milder wilting, although mycelial growth is not prevented [20]. More holistic disease management approaches have also been proposed but *Fol* continues being a serious threat to tomato production in many areas of the world [21]. Such a daunting scenario has spurred an intense quest for molecular sources of resistance.

Previous transcriptome studies in different plants infected with *F. oxysporum* have revealed quite variable transcriptional responses ostensibly dependent on plant species and *F. oxysporum* strain combinations. For example, in the model plant *A. thaliana* a series of upregulated genes have been suggested as a *b Bona fide* defense response against the fungus [22]. In two cultivars of flax with different susceptibility to *F. oxysporum* f. sp. *lini*, it was shown that the most resistant cultivar deployed a defense response that included WRKY transcription factors, ethylene regulators, and flavonoid-related enzymes, among others [23]. In a resistant cultivar of *Medicago trucantula* infected with *F. oxisporum* f. sp. *medicaginis,* genes encoding proteins related to sugar, protein, cell wall metabolism, nutrients uptake and oxidative processes were found enriched [24]. In highly resistant plants of *B. oleracea* infected with *F. oxysporum* f. sp. *conglutinans* early plant defense responses included MAPK signaling, calcium signaling, and ROS induction. In addition, pathogenesis-related (PR) proteins, ABC transporters and several transcription factors were activated [25]. Two micro (mi)-RNAs, slmiR482f and slmiR5300, were found downregulated in the Motelle tomato cultivar upon *Fol* infection. Those miRNAs were proposed to act as constitutive repressors of four uncharacterized proteins harboring nucleotide binding domains with putative function in anti-fungal immunity [26]. Finally, in tomato plants susceptible or resistant to *Fol*, it was found that the incompatible interaction established in the resistant cultivar was accompanied by secondary metabolite production and tryptophan metabolism [27].

Because the molecular interaction between *Fol* and tomato cultivars growing in Colombian fields remained unexplored, we decided to conduct whole-genome transcriptome analysis of plants infected with either a pathogenic (*Fol*-pt) or a non-pathogenic (*Fo*-npt) strain of *Fo* in two commercial and one wild tomato cultivars common in the department of Caldas, Colombia. Such commercial cultivars were chosen based on a prior survey conducted with tomato growers, about their cultivar preferences in the Caldas region. We hypothesized that tomato varieties mounting an effective defense response will overexpress resistance-associated genes that could be used in traditional genetic breeding or assayed in transgenesis experiments. Our results suggest that the cultivars analyzed exhibit a differential molecular response to *Fol* infection and differentially expressed genes might constitute the foundations for genetic breeding programs against *Fol* in Colombia.

## Results

**Fig. 1:**
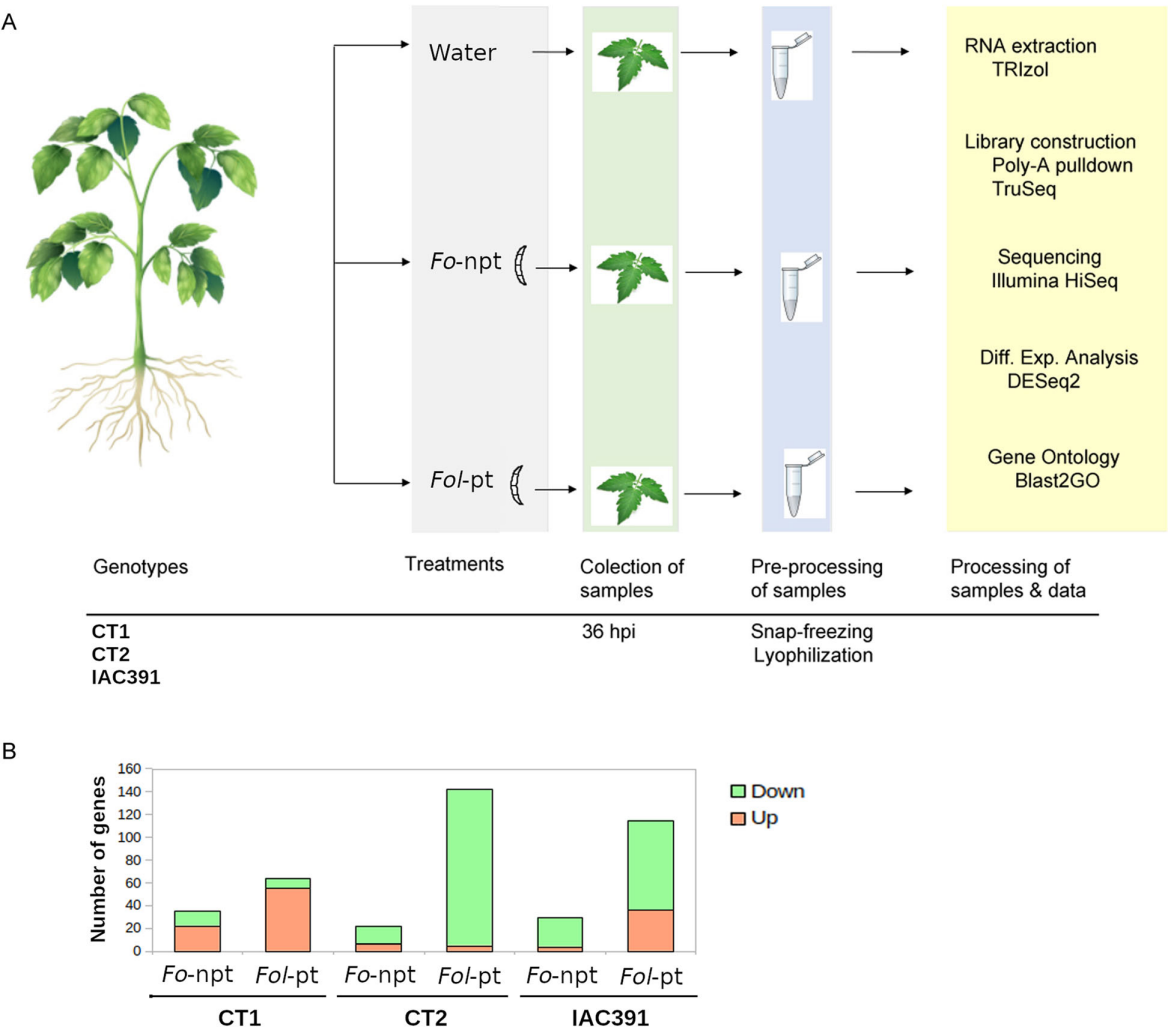
**A** Description of experiments. Plants of the cultivars CT1 or CT2, as well as the wild genotype ICA391 were mock-inoculated or inoculated with either a non-pathogenic (*Fo*-npt) or a pathogenic (*Fol*-pt) strain of *Fusarium oxysporum* f. sp*. lycopersici*, at 30 days post-germination. Two biological replicates were included per cultivar. Inoculation was on the soil-stem junction without mechanical damage. Systemic leaves of inoculated plants were collected 36 h post-inoculation (hpi). Collected leaves appear encircled. Leaves were snap-frozen in liquid nitrogen upon collection and subsequently lyophilized. Lyophilized material was used for RNA extraction and construction of RNAseq libraries. **B** Number of differentially expressed genes in each tomato cultivar inoculated with the pathogenic (*Fol*-pt) or non-pathogenic (*Fo*-npt) strain of *Fol*

To characterize the molecular response of three cultivars of tomato (CT1, CT2 and IAC391) regarded as resistant to infection by *Fusarium oxysporum* f. sp. *lycopersici* (*Fol*) in the Caldas department of Colombia, we conducted transcriptomic analysis by RNAseq, upon infection of tomato plants with either a non-pathogenic or a pathogenic strain of *Fol*. For the sake of simplicity, we refer to those strains as *Fo*-npt and *Fol*-pt, respectively. Details of our inoculation experiment are graphically depicted in Fig. 1A. It is important to clarify that we decided to take samples at 36 h post-inoculation (hpi) because we were interested in an early defense response of tomato plants against *Fol*. Moreover, distal (upper) leaves were sampled because our interest was in the systemic molecular defense responses induced by *Fol* in tomato cultivars. The use of a non-pathogenic strain of *Fo* is an additional control that allowed us to focus on bona fide defense-related genes. Early defense-response genes have the potential to be used in genetic breeding programs aimed at conferring resistance against *Fol*. Hereafter, we present the results per cultivar (Fig. 1B). Because the transcriptome of uninfected tomato plants between cultivars is substantially different (Supp. Figure 1), in each experiment, we used control plants belonging to the same cultivar inoculated with *Fo*-npt or *Fol*-pt. In general, the overlap between genes differentially expressed in each cultivar was very scarce, and so was among plants inoculated with either *Fo*-npt or *Fol*-pt (Supp. Figure 2).

### Commercial tomato 1 (CT1)

**Fig. 2:**
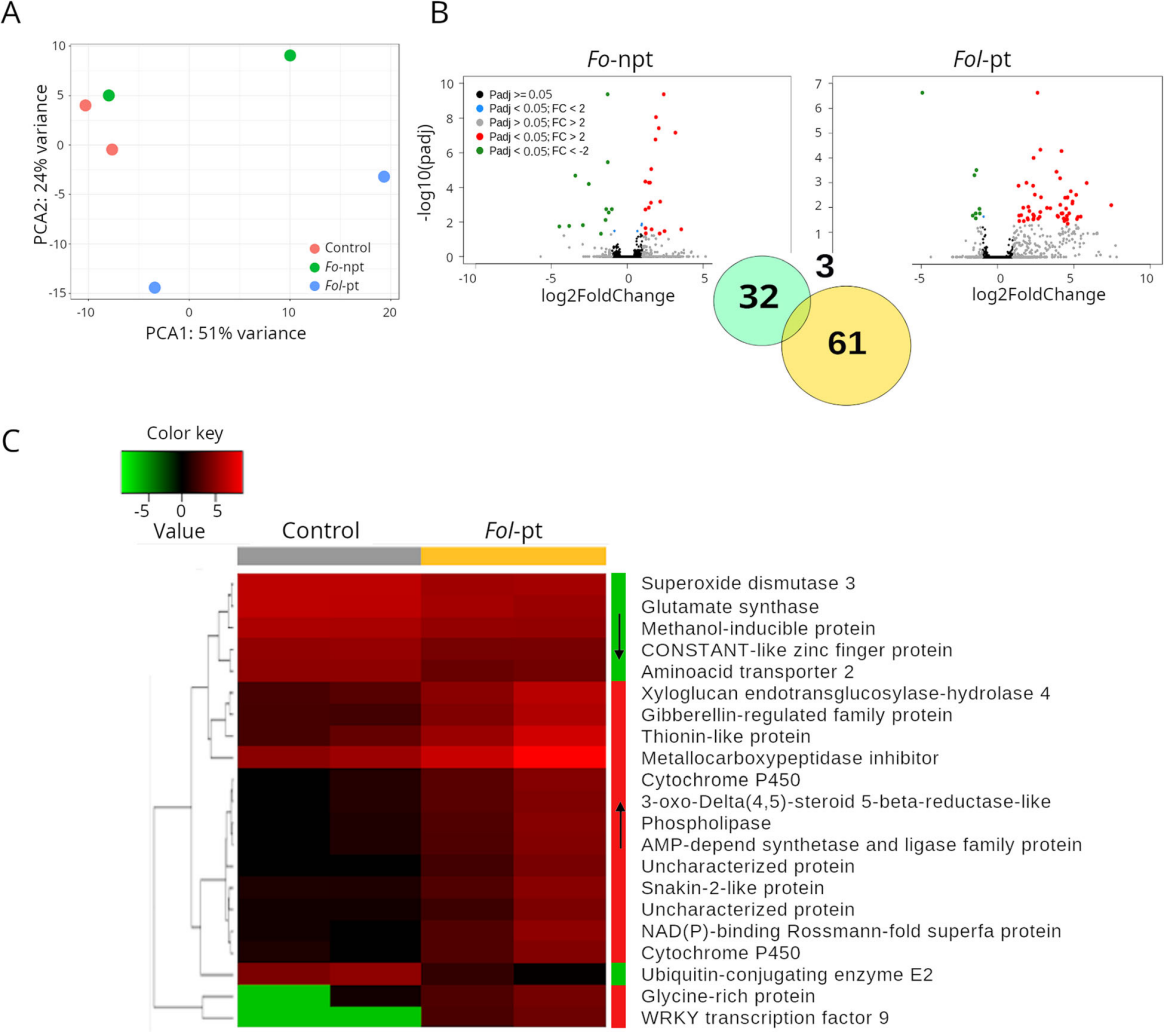
RNAseq results for cultivar CT1. **A** Principal component analysis (PCA) shows that the overall transcriptional profile of plants infected with the non-pathogenic strain of *Fol* is more similar to that of mock-inoculated plants, suggesting that such difference is due to the pathogenic infection. **B** Volcano plots depicting differentially expressed genes in plants infected with the non-pathogenic (left panel) or the pathogenic (right panel) strains of *Fol*. As inset, a Venn diagram showing the number of genes uniquely differentially expressed for each strain or common to both strains are shown. **C** Heatmap depicting the normalized expression of the top 25 differentially expressed genes. Normalized data is presented in a logarithmic scale. Arrows on the side color bars of the heatmap indicate blocks of genes that were downregulated (downward arrow) or upregulated (upward arrow) in plants treated with *Fol*-pt

For plants inoculated with *Fo*-npt (Supplementary Table S1), the overall transcriptional profile exhibited a closer resemblance to the profile of mock-inoculated control plants than to the one of plants inoculated with *Fol*-pt (Fig. 2A; Supplementary Table S2). This suggests that the transcriptomic changes observed in tomato plants inoculated with *Fol*-pt are likely due to the pathogenic infection of the fungus since *Fol*-pt samples clearly separated from control and *Fo-*npt samples in a principal component analysis (PCA) plot (Fig. 2A). Substantial variability between infected plants was also observed (Fig. 2A). In differential expression analysis between control plants and those inoculated with *Fo*-npt, 35 genes were found differentially expressed (22 and 13 upregulated and downregulated genes, respectively) (Fig. 2B, left panel; Supplementary Table S1). By contrast, 64 genes were found differentially expressed in plants infected with *Fol*-pt (55 and 9 upregulated and downregulated genes, respectively) (Fig. 2B, right panel; Supplementary Table S2). The fold change of upregulated genes in the case of *Fol*-pt was substantially higher than the corresponding one for *Fo*-npt (average 18 and 3.8, respectively). Overlap between genes differentially expressed by *Fo*-npt and *Fol*-pt was rather scarce. Thus, a more robust transcriptional response was elicited by *Fol*-pt. Only three genes were differentially expressed in both datasets (Solyc05g055330, Solyc10g075100 and Solyc11g013810) (Fig. 2B, inset). The first two were upregulated in both cases and encode a drug resistant transporter ABC-like protein and a non-specific lipid transfer protein. The third gene was downregulated in both cases and encodes a nitrate reductase. Such a poor overlap suggests that genes differentially expressed only by *Fol*-pt might be associated with disease production. Because anti-fungal defense includes the induction of expression of defense-related genes [28] we focused on the set of genes that were found upregulated in plants inoculated with *Fol*-pt, which were the majority. Those genes have the potential to be used in genetic breeding programs. Several genes known to have a role in anti-fungal defense were found upregulated. For instance, two WRKY transcription factors (Solyc01g104550 and Solyc02g080890) were found upregulated 175-fold and 7-fold, respectively. WRKY transcription factors have been reported to play a role in responses to abiotic and biotic stresses [29] and more specifically in plant immunity [30]. In addition, five genes encoding cytochrome P450 (CYP) proteins were found upregulated with fold-change that ranged from 4 to 30. CYP proteins have been implicated in a diverse array of defense responses by regulation of the synthesis of terpenes [31]. A conspicuously upregulated gene was an acidic extracellular 27kD chitinase (Solyc02g082930; fold-change = 5.03), which is a gene directly implicated in anti-fungal defense [32]. The expression profile of the top differentially expressed genes in this cultivar is presented in Fig. 2C.

To gain additional insights into the physiological processes and functions potentially affected by differentially expressed genes, we conducted gene ontology analysis (GO) using Blast2GO [33], only for genes upregulated during infection with *Fol*-pt. Fifteen upregulated genes were mapped to the oxidation-reduction process (P:GO:0055114), including all cytochrome P450 genes, dioxygenases, dehydrogenases, among others (Supplementary Table S7). Other ostensibly defense-related processes or functions identified on the basis of the upregulated genes included metal ion binding (F:GO:0046872), terpenoid biosynthetic process (P:GO:0016114) and regulation of transcription DNA-templated (P:GO:0006355), represented by the WRKY transcription factors differentially expressed. Thus, the induction of defense related genes was the predominant response observed in the cultivar CT1 upon infection with *Fol*-pt.

### Commercial tomato 2 (CT2)

**Fig. 3:**
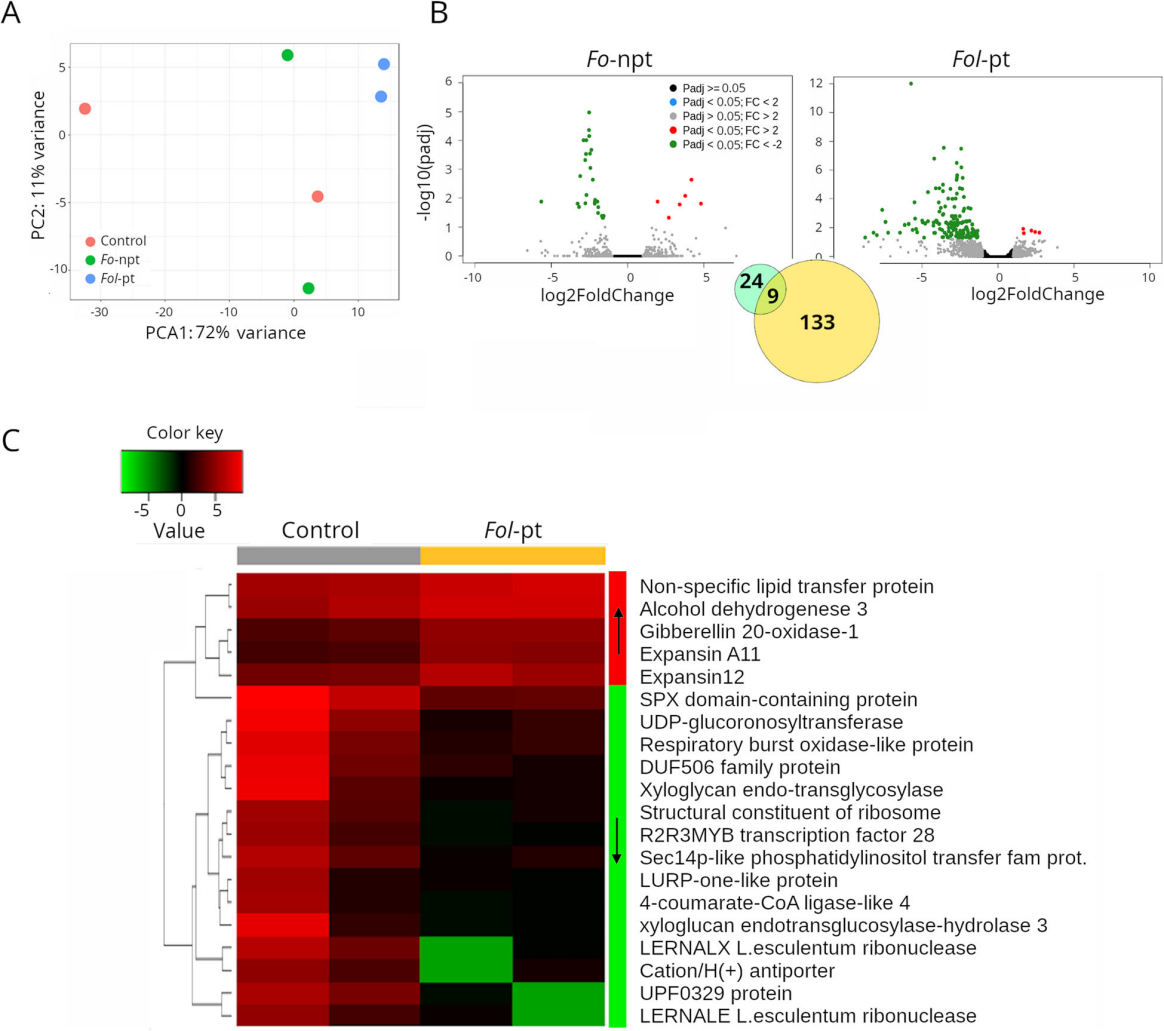
RNAseq results for cultivar CT2. **A** Principal component analysis (PCA) shows that the overall transcriptional profile of plants infected with the non-pathogenic strain of *Fol* is more similar to that of mock-inoculated plants, suggesting that such difference is due to the pathogenic infection. **B** Volcano plots depicting differentially expressed genes in plants infected with the non-pathogenic (left panel) or the pathogenic (right panel) strains of *Fol*. As inset, a Venn diagram showing the number of genes uniquely differentially expressed for each strain or common to both strains, is shown. **C** Heatmap depicting the normalized expression of the top 25 differentially expressed genes. Normalized data is presented in a logarithmic scale. Arrows on the side color bars of the heatmap indicate blocks of genes that were downregulated (downward arrow) or upregulated (upward arrow) in plants treated with *Fol*-pt

The transcriptome of plants infected with *Fol*-pt (Supplementary Table S4) was clearly different from mock-inoculated plants or from plants infected with *Fo*-npt (Supplementary Table S3). Of note, mock-inoculated and *Fo*-npt-inoculated plants were located close to each other in a PCA plot, while plants infected with *Fol*-pt clearly separated from the rest but by comparison to CT1 (Fig. 2A), they were located closer to the other two groups (Fig. 3A). In a way, this suggests that the transcriptional response to *Fol* infection in the CT2 cultivar was milder than in the previous case, because *Fo*-npt-inoculated plants could not be clearly separated from control plants based on their Euclidean distances. Indeed, the transcriptional response to *Fol* infection in CT2 was substantially different to the one seen in CT1. The most conspicuous difference was the predominance of downregulated genes. Namely, in the case of *Fo*-npt, 16 and 6 genes were found downregulated and upregulated, respectively. Similarly, for *Fol*-pt, 137 and 5 genes were found down- and up-regulated, respectively. Nine genes were found down-regulated in both the *Fo*-npt and *Fol*-pt experiments (Fig. 3B). In plants infected with *Fol*-pt, a series of transcription factors, phosphatases and kinases were included among down-regulated genes. The expression profile of the top differentially expressed genes in this cultivar is presented in Fig. 3C.

We also conducted gene ontology analysis on the down-regulated genes (Supplementary Table S8). Enriched gene ontology terms included ATP binding (F:GO:0005524), metal ion binding (F:GO:0046872), oxidation-reduction process (P:GO:0055114), regulation of transcription (P:GO:0006355), defense response (P:GO:0006952), and RNA binding (F:GO:0003723). It is possible that the downregulation of transcription factors and RNA-binding proteins account for the overall downregulation of genes observed in this variety. At least at the molecular level, this cultivar should be considered permissive to *Fol* infection. Complementary studies including disease severity and reduction of yield are necessary to declare this variety as susceptible.

### IAC391

**Fig. 4:**
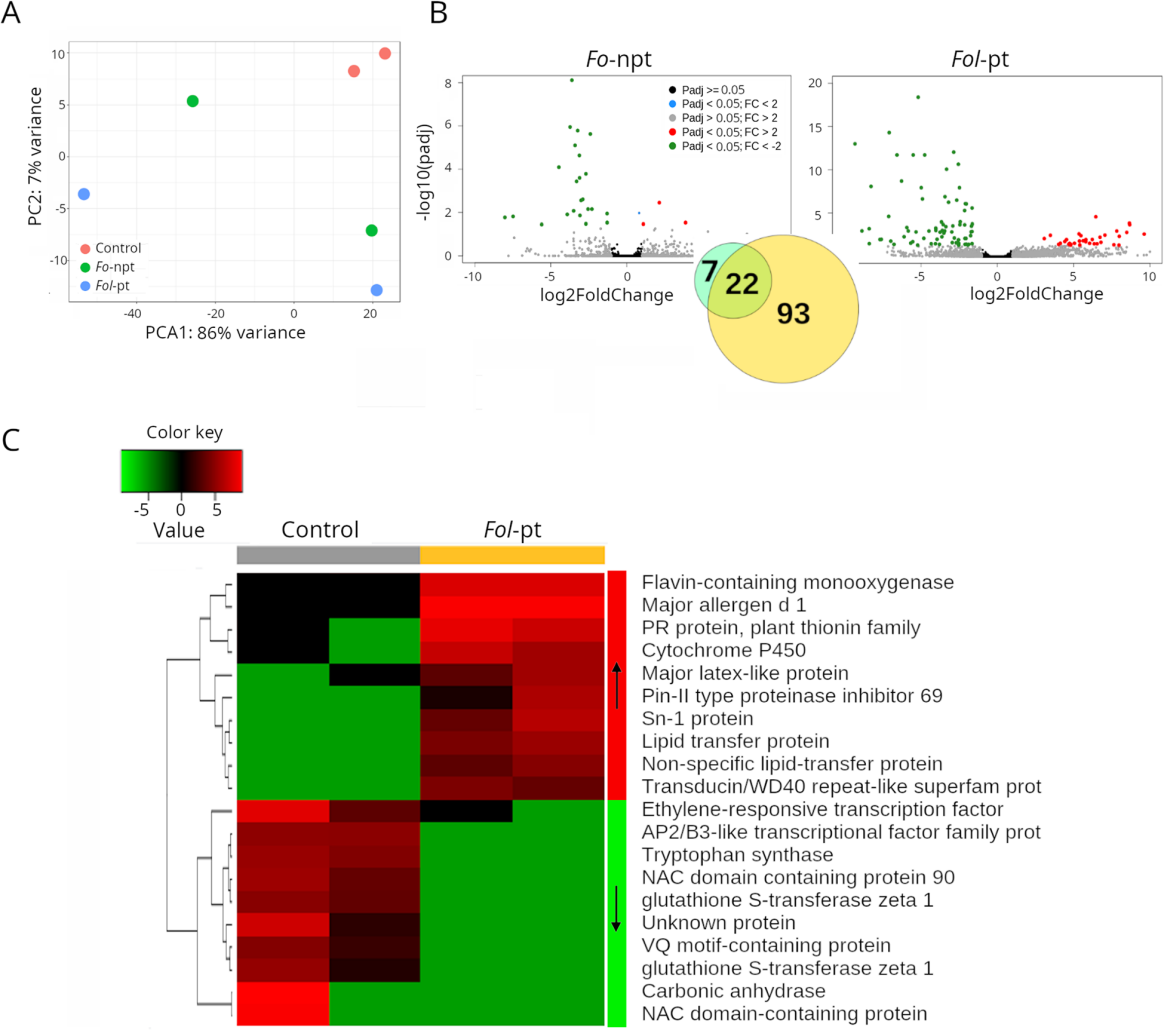
RNAseq results for cultivar IAC391. **A** Principal component analysis (PCA) shows that the overall transcriptional profile of plants infected with the non-pathogenic strain of *Fol* is more similar to that of mock-inoculated plants, suggesting that such difference is due to the pathogenic infection. **B** Volcano plots depicting differentially expressed genes in plants infected with the non-pathogenic (left panel) or the pathogenic (right panel) strains of *Fol*. As inset, a Venn diagram showing the number of genes uniquely differentially expressed for each strain or common to both strains, is shown. **C** Heatmap depicting the normalized expression of the top 25 differentially expressed genes. Normalized data is presented in a logarithmic scale. Arrows on the side color bars of the heatmap indicate blocks of genes that were downregulated (downward arrow) or upregulated (upward arrow) in plants treated with *Fol*-pt

In the IAC391 cultivar, the transcriptome of plants inoculated with either *Fo*-npt (Supplementary Table S5) or *Fol*-pt (Supplementary Table S6) was clearly distinct from the transcriptome of mock-inoculated plants (Fig. 4A). The transcriptional response observed in IAC391 was much more complex than the one observed in the two other cultivars. In plants infected with *Fo*-npt, 4 and 25 genes were found up- and downregulated, respectively (Fig. 4B; Supplementary Table S5). Among the four upregulated genes, the proteins pathogenesis-related 5-like protein (Solyc08g080670), diacylglycerol kinase 5 (Solyc08g082190) and an ethylene forming enzyme (Solyc12g098850) are encoded, presumably with a role in defense against pathogen attack. Interestingly, several other genes encoding defense-related proteins were found downregulated. Plants infected with *Fol*-pt, as before, exhibited a much more robust transcriptional response. Respectively, 36 and 79 genes were found up and downregulated (Supplementary Table S6). Upregulated genes included pathogenesis-related proteins, cytochrome P40 proteins and terpenes, which are genes with potential roles in defense. Downregulated genes also included pathogenesis-related proteins and cytochrome P450 proteins, among many others (Supplementary Table S6). The expression profile of the top differentially expressed genes in this cultivar is presented in Fig. 4C.

Because a considerable number of genes were found up- and downregulated in IAC391, gene ontology analysis was conducted on both set of genes. For upregulated genes (Supplementary Table S9), as in the case of CT1, there was an enrichment of the oxidation-reduction process (P:GO:0055114), response to biotic stimulus (P:GO:0006355) and regulation of transcription (F:GO:0016717). Interestingly, the oxidation-reduction process was associated with downregulated genes too (Supplementary Table S10). Remarkably, the defense response GO process (P:GO:0006952) was associated with 12 genes exhibiting drastic downregulation. Metal ion binding (F:GO:0046872) and lipid transport (P:GO:0006869) were also found enriched by downregulated genes. In other words, the fact that genes and gene ontology terms involved in defense responses were found up- and downregulated points toward a genuine arms race between pathogenicity factors of the fungus and defense mechanisms of the plant in this cultivar.

### Distribution of differentially expressed genes along chromosomes

**Fig. 5:**
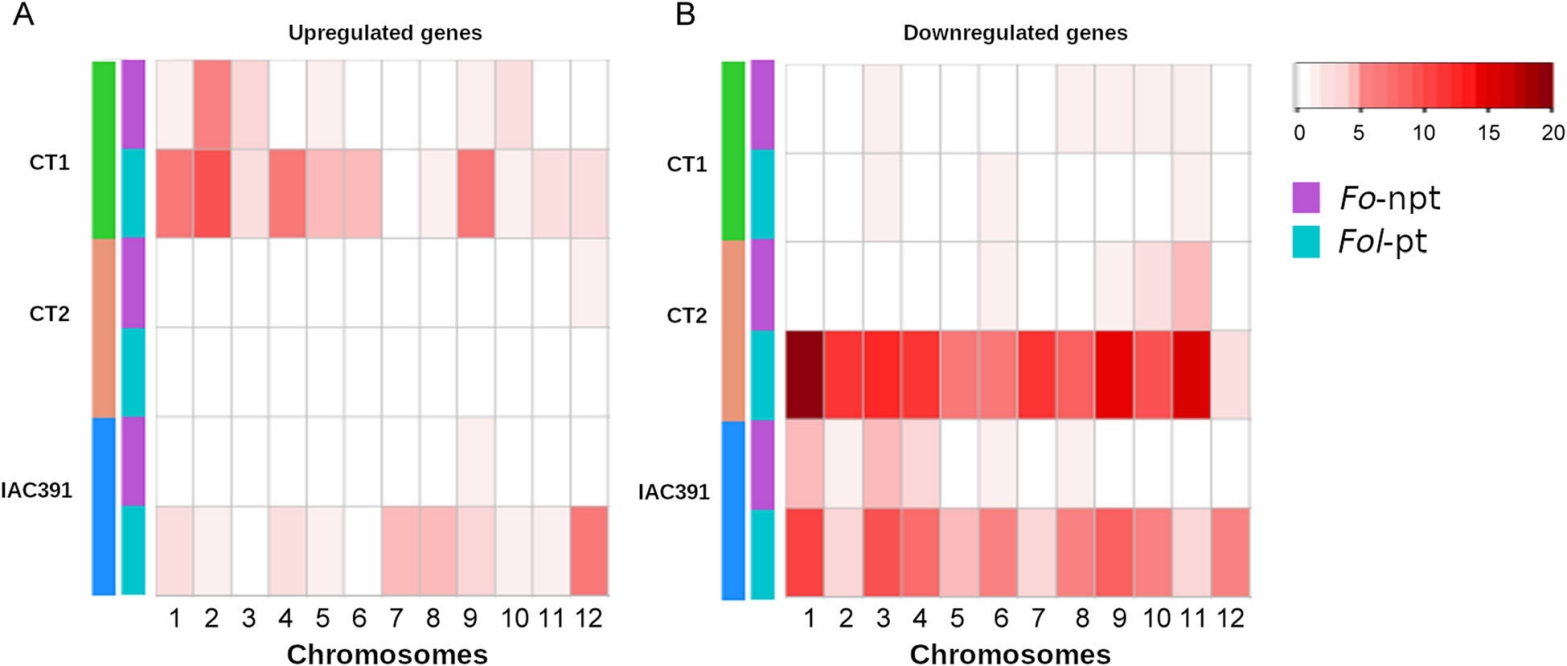
Number of differentially expressed genes per chromosome. Location along chromosomes was determined during alignments. The name of cultivars and strain of *Fol* are indicated. **A** Upregulated genes. **B** Downregulated genes

Differentially expressed genes mapped to all chromosomes, but its location was divergent among cultivars. In Fig. 5, the number of genes per chromosome was plotted on a heatmap, using the same intensity scale (0–20), so that intensities are comparable between samples, between fungus isolates, and between upregulated (Fig. 5A) and downregulated (Fig. 5B) genes. For upregulated genes, the number of genes induced by *Fol*-pt was clearly higher than the ones induced by *Fo*-npt, especially in the cultivars CT1 and IAC391, but they showed a distinct spatial distribution along chromosomes. The higher number of upregulated genes in CT1 clustered on chromosomes 1,2,4 and 9, while in IAC391 they clustered on chromosomes 7,8 and 12. The number of upregulated genes in CT2 was very low and similar for *Fol*-pt and *Fo*-npt (6 and 5 genes, respectively). For downregulated genes, CT1 registered few genes, for both *Fol*-pt and *Fo*-npt. CT2 and IAC391 both registered many downregulated genes and their distribution pattern along chromosomes was similar, but clearly distinct. Namely, downregulated genes in the CT2 were in all chromosomes but mainly in chromosomes 1, 2, 3, 4, 7, 9, and 11; while in IAC391 they were mostly on chromosomes 1, 3, and 9 (Fig. 5)

## Discussion

At the molecular level, imbricated interactions between effector molecules from phytopathogenic fungi and the host plant defense machinery take place, which results in either disease or resistance [19]. We conducted RNAseq in three different Colombian tomato cultivars to portray the molecular response to infection by a pathogenic (pt) or a non-pathogenic (npt) strain of *Fusarium oxysporum*. In all cases, the pathogenic strain induced more robust transcriptional changes, which we assume occurred in response to the infective process promoted by *Fol*-pt. Because our interest is to determine molecular changes associated with disease production, we discuss the response of tomato plants to *Fol*-pt. It is important to mention here that the number of differentially expressed genes in our study was relatively small, but our experiments, specially the sampling time at 36 h post-inoculation were precisely intended to detect early defense responses against *Fol*. This is based on the premise that, for a source of resistance to be effective against a pathogen, it should act as early as possible during infection.

### Induction of defense-related genes during *Fol* infection

The response of the cultivar CT1 to infection with *Fo*-npt or *Fol*-pt consisted predominantly of the upregulation of gene expression; many of those genes are involved in defense. For instance, upregulation of WRKY transcription factors 6 and 9 (Solyc02g080890 and Solyc01g104550; Table 1), was observed only in the presence of the pathogenic strain of *Fol*. This is consistent with a recent study, using microarrays, wherein 16 different WRKY transcription factors were found upregulated in tomato plants infected with *Fol*, among which WRKY36 and WRKY37 stood out [29]. Thus, it is possible that the plethora of WRKY transcription factors represents the evolution of specialized responses against different *Fol* strains [34, 35], and more generally, against different pathogens [30]. WRKY transcription factors were not found upregulated in the cultivars CT2 or IAC391.

**Table 1:** Representative genes differentially expressed by infection of *Fol*-pt and gene ontology terms associated

Gene	Log2 Fold-change	Adj. p-value	Go term	Gene description
			**CT1**	
Solyc02g080890	2.80907115	4.6862E-05	F:GO:0003700	WRKY transcription factor 6
Solyc01g104550	7.44730817	0.00806559	F:GO:0003700	WRKY transcription factor 9
Solyc02g082930	2.33107546	0.00131687	F:GO:0004568	Acidic extracellular 27 kD chitinase
Solyc11g013110	1.89560049	0.00101235	F:GO:0004553	Flavonol synthase
Solyc03g115220	1.55351214	0.02040609	F:GO:0005506	Flavonoid 3′-hydroxylase
Solyc12g042500	4.69099536	0.01727904	–	Gibberellin-regulated family protein
Solyc12g042520	4.49358815	0.01104915	–	Gibberellin-regulated family protein
Solyc06g059930	4.1876883	5.2556E-05	F:GO:0000287	Sesquiterpene synthase 1
Solyc01g105880	3.9202373	6.067E-05	F:GO:0000287	Monoterpenoid synthase 2
			**CT2**	
Solyc01g096190	−2.2633495	0.01505172	F:GO:0005388	Ca2 + −ATPase
Solyc01g058720	−3.4691547	0.00034242	F:GO:0005509	Calcium-binding EF-hand
Solyc06g065820	−4.6022105	0.00366324	F:GO:0003677	Ethylene-respons transcript factor ERF003-like
Solyc11g012980	−4.8573007	0.04893111	F:GO:0003677	Ethylene-respons transcript factor ERF014-like
Solyc10g079860	−3.8592592	0.00552977	F:GO:0005506	L.esculentum TomQ’b beta(1,3)glucanase
Solyc03g006880	2.44990544	0.01969419	–	Gibberellin 20-oxidase-1
			**IAC391**	
Solyc01g091160	4.81325521	0.03935622	F:GO:0004053	Pathogenesis-related 5-like protein
Solyc10g080010	4.53194493	0.01116834	F:GO:0016757	Geranylgeranyl pyrophosphate synthase 1
Solyc07g066330	3.07847198	0.00853798	F:GO:0003677	Sesquiterpene synthase
Solyc02g071475	5.48144135	0.00393161	F:GO:0008168	Cytochrome P450, family 81
Solyc10g005320	9.62306957	0.0025659	F:GO:0004834	Tryptophan synthase beta chain 1-like
Solyc07g044900	8.67617934	0.00021562	–	Tryptophan synthase
Solyc03g114540	−4.9058111	1.9386E-10	F:GO:0016844	Sn-1 protein
Solyc07g055950	−5.3240287	3.7529E-06	C:GO:0016021	Sn-2 protein
Solyc08g066260	−6.5689437	1.9717E-12	F:GO:0004398	Rapid alkalinization factor 3

A conspicuous upregulated gene in CT1 was the acidic 27 kDa endochitinase (CHI17, Solyc02g082930; Table 1) which is involved in defense against chitin-containing fungal pathogens like *Fol* [36]. According to the STRING protein database (string-db.org) this chitinase interacts with several pathogenesis-related (PR) proteins. Similarly, pathogenesis-related protein 5-like protein (Solyc01g091160; Table 1) was induced in IAC391, although it was also induced by *Fo*-npt, suggesting that it is perhaps involved in basal defense. This protein belongs to the thaumatin family of PR proteins involved in defense response to biotic factors [37], including fungal pathogens [38]. No gene involved in a defense response was found upregulated in CT2.

Genes related to the synthesis of flavonoids were found upregulated in CT1 (Solyc11g013110, Solyc03g115220) and IAC391 (Solyc10g080010, Solyc07g066330), but not in CT2 (Table 1). Flavonoids synthesis is activated upon pathogen attack and can inhibit microbial cellulases, pectinases and xylanases [39]. Gibberellins are closely related to plant defense responses against pathogens [40], and they were found upregulated in CT1 (Solyc12g042500, Solyc12g042520) and CT2 (Solyc03g006880) (Table 1). Induction of genes associated with gibberellins has been reported in melon and chickpea plants infected with different species of *Fusarium* [41, 42]. Genes associated with the synthesis of terpenes were also found upregulated in CT1 (Solyc06g059930, Solyc01g105880) and IAC391 (Soly10g080010, Soly07g066330) (Table 1). Terpenes are synthesized either through the mevalonate or the MEP pathways and have been implicated in defense to microbes and insects [43–46]. Volatile compounds serve as an alert strategy among plants to prime systemic defense responses, which increase tolerance to environmental conditions. A few genes encoding mono-oxygenases in the family cytochrome P450 (CYPs) were found upregulated in CT1 and one in IAC391 (Solyc02g071475) (Table 1). CYP enzymes are involved in redox reactions and biosynthesis of compounds like fatty acids, alkaloids, flavonoids and other secondary metabolites like phytoalexins [31]. Overexpression of CYP genes has been reported in potato plants in response to *Phytophthora infestans* infection [47]. However, it should be mentioned that CYP genes were also found upregulated in plants inoculated with *Fo*-npt, which hints to a general defense response independent of pathogen virulence genes. Two genes associated with the synthesis of tryptophan (Solyc10g005320, Solyc07g044900; Table 1) were found upregulated in IAC391. This amino acid is involved in the hypersensitive response to hemi-biotrophic pathogens like *Bipolaris oryzae* in rice [48] and *Verticillium longisporum* [49], *Colletotrichum gloesporiodes* [50] and *Fusarium* spp. [51] in Arabidopsis.

### Genes repressed during *Fol* infection

Although all tomato cultivars underwent downregulation of some genes upon *Fol* infection, such phenotype was notably stronger in CT2. For example, genes associated with the synthesis and transport of calcium (Solyc01g096190, Solyc01g058720; Table 1) were found downregulated in CT2. It has been reported that calcium signaling plays an important role in effector-triggered immunity (ETI) in response to pathogen-associated molecular patterns (PAMPs) [52]. Likewise, two ethylene-responsive transcription factors (Solyc06g065820, Solyc11g012980; Table 1) were found downregulated. These genes belong to a family of transcription factors important for regulation of ethylene, and their depletion should negatively impact ethylene’s role in pathogen defense and ROS response [53, 54]. A glucanase (Solyc10g079860) was also found downregulated in CT2 (Table 1). Glucanases belong the PR2 protein family and have antifungal activity by themselves or in association with chitinases and other antifungal proteins [55].

Most differentially expressed genes in IAC391 were downregulated. Among them are Sn-1 and Sn-2 (Solyc03g114540 and Solyc07g055950; Table 1), which are peptides involved in defense against pathogens in potato [56, 57] and tomato [58, 59]. Moreover, Rapid Alkalinization Factor 3, RALF (Solyc08g066260; Table 1), which is involved in plant immune responses, was also found downregulated. RALF has also been reported downregulated in chickpea roots infected with *F. oxysporum* f. sp. *cicero* Race 1 (*Foc* Race 1) [60]. It has been shown that tomato roots colonized by *F. oxysporum* undergo alkalization, which contributes to activation of essential mitogen-activated protein kinase Fmk1, important for pathogen colonization [61]. The cultivar CT1 exhibited only few downregulated genes with no evident role in disease.

In summary, the transcriptional response of each cultivar to *Fol* infection had a unique profile, evidencing that the genome of tomato encodes a plethora of mechanisms that are partially expressed in each case, perhaps depending on the specific interactions of each cultivar with *Fol* races and environmental conditions. The response of CT1 seems in line with resistance, because the expression of defense genes was actively induced, including a chitinase and WRKY transcription factors. We summarize the transcriptional response of CT1 as a remarkable upregulation of genes. Growers regard CT1 as a cultivar resistant to *Fol*. On the contrary, CT2 exhibited predominat downregulation of genes, including transcription factors, phosphatases and kinases. This could be interpreted either as the shutdown, by the host, of factors that favor fungal pathogenicity or as the inactivation of defense responses by the fungus. Because the local growers also regard CT2 as a resistant variety, we favor the first hypothesis, but clearly more investigation is needed. Lastly, IAC391 exhibited a somewhat intermediate response, although its transcriptional response was predominantly downregulation of gene expression, it also exhibited a considerable number of genes upregulated, which perhaps evidences an arms race between such accession and *Fol*. IAC391 is considered a wild genotype, although experimentally is cultivated with satisfactory performance in terms of yield of commercial fruits [59, 60] More in-depth studies, including agronomic evaluation of cultivars exposed to *Fol* will provide additional insights into more subtle differences in resistance or tolerance observed in each cultivar.

### Chromosomal localization of differentially expressed genes in response to *Fol* infection

Distribution of differentially expressed genes along tomato chromosomes was quite variable among cultivars. Downregulated genes, especially in the case of *Fol*-pt, were more abundant on chromosomes 3, 6 and 11, in the three cultivars. CT2 and IAC391 were more similar in this respect, with numerous downregulated genes in most chromosomes. Conversely, for upregulated genes, the response of CT1 and IAC391 was more similar, although considerably discordant in the spatial distribution of genes along chromosomes. CT2 showed only a handful of upregulated genes. Taken together, the spatial distribution of differentially expressed genes in the different cultivars suggests that the genetic and/or epigenetic defense strategies of each cultivar against *Fol* are not only qualitatively but also structurally distinct, which may point to distinct processes of coevolution with *Fol*. However, we do acknowledge that such divergent spatial distribution of differentially expressed genes along the genome might reflect, to some extent, chromosomal rearrangements of each genome during natural evolution and/or genetic breeding [62, 63].

In summary, some of the genes found upregulated in this study are interesting candidates to transfer resistance to susceptible cultivars with other desired agronomic traits like yield, flavor or color, but susceptible to *Fol*. Examples include pathogenesis-related 5-like protein (Solyc08g080670), diacylglycerol kinase 5 (Solyc08g082190) and ethylene forming enzyme (Solyc12g098850) found upregulated in IAC391 and acidic 27 kDa endochitinase (CHI17, Solyc02g082930) found upregulated in CT1.

## Conclusion

Selection of tomato cultivars in Colombia is made essentially based on recommendations of commercial entities that import and distribute seeds. Although such cultivars may have been evaluated for resistance against pathogens in other countries, it does not guarantee resistance against local biotic and environmental conditions. We therefore recommend that introduction of new cultivars should be anteceded by whole transcriptome analyses like the one presented here, whenever it is possible. In this particular case, we would recommend the cultivars CT1 or IAC391, given that their transcriptional response was more in line with a resistant phenotype. A limitation of our study was that experimental plants were not evaluated after sampling of systemic tissue for RNAseq analysis, which prevented observation of symptoms severity and assessing the impact of infection on plants’ yield. Obviously, transcriptome analyses should be complemented with pathogenicity field experiments evaluating crop yield and inoculum abundance of *Fol* in each cultivar.

## Methods

### Isolation and activation of the fungus

*Fusarium oxysporum* f. sp. *lycopersici* (*Fol*) strains used in this study were provided by the laboratory of Plant Pathology of the Universidad de Caldas. The pathogenic (*Fol-UDC10,* Race 2*)* and non-pathogenic isolates (*Fol-UDC7)* [64] were isolated from tomato fields of the Caldas department. Both isolates were reactivated in 30-day-old seedlings of the cultivar IAC391 produced in vitro. Once symptoms were observed, infected plant tissue was cultured on PDA medium (39 g.L^− 1^ of water) supplemented with 3.9 g.L^− 1^ extracts of macerated roots of IAC391 seedlings, to promote fungus growth. Plants were incubated for 10 days at 27 °C, in darkness. The presence or absence of virulence genes in each isolate were verified by PCR [64].

### Collection of macro- and microconidia

After sporulation was observed, five confluent Petri dishes were washed with 4 mL of distilled water for each of the isolates. Conidia were counted in a hemocytometer and diluted to a concentration of 1 × 10^6^ conidia/ml. This was the inoculum. A day before inoculations in the field, a pathogenicity test was carried out in vitro, inoculating IAC391 seedlings with 10 μL of the conidial suspension and incubating them at 28 °C for 1 week. Disease symptoms were then evaluated to confirm that the inoculum used in the field was viable.

### Field infection experiments

Infection experiments were conducted in the farm Montelindo property of Universidad de Caldas. Such farm is located in Santágueda, in the Palestina municipality in the department of Caldas, at 1050 m.a.s.l, with an annual average temperature of 28 °C, relative humidity of 76% and annual precipitation of 2100 mm. Average temperature during the experiments period was 28 °C. Seedbeds were prepared using the soil fumigant dazomet (Basamid® GR). Thirty days after germination, seedlings were transplanted to individual 5 kg plastic bags filled with sterilized soil. Plants were placed onto 50 cm-tall benches inside a greenhouse with restricted access, to prevent cross-contamination. Plant material were two commercials (CT1, CT2) and one wild (IAC391) cultivars known to be resistant to *Fol* infection but with unknown genetic background. Management of plants was as is conventional in this zone. For each cultivar: Commercial Tomato 1 (CT1), Commercial Tomato 2 (CT2), both regarded as resistant to *Fol* race 2 and IAC391 (with unknown response to *Fol*); five plants were inoculated with 75 mL of the conidial suspension (1 × 10^6^ conidia/ml) of the pathogenic isolate (*Fol*-pt), five with the non-pathogenic isolate (*Fo*-npt) and five were mock-inoculated with water. Inoculum was administered to the soil, at the stem-soil junction, without causing any damage to the stem. Two different experiments were conducted. Initially the cultivar IAC391 was evaluated. Based on results derived from this experiment, the two commercial cultivars frequently planted in the zone were selected for a second round of experiments. For RNAseq analysis, two (2) plants from each treatment were selected randomly, and from each of them ca. 100 mg of systemic leaves were collected in darkness, 36 h post inoculation. This short time until sampling was intended to capture early plant defense responses induced by *Fol*. Collected material was snap-frozen in liquid nitrogen, and subsequently lyophilized and sent to NOVOGENE, Hong Kong for RNA extraction, library construction and sequencing.

### Library construction and sequencing

Total RNA was extracted with TRIzol reagent (Invitrogen). RNAseq libraries were constructed from 500 ng of total RNA using the NEBNext Ultra II Directional RNA Library Prep Kit for Illumina (NEB). Polyadenylated mRNAs were enriched with oligo dTs conjugated to paramagnetic beads. Enriched mRNAs were fragmented chemically and used for cDNA synthesis. cDNA was end-repaired and A-tailed, ligated to linkers and finally indexed by PCR, to enable multiplexing during sequencing. Sequencing was done on a HiSeq2500 instrument, following a paired-end 150 cycles protocol. Average sequencing depth was 12 million paired-end reads per sample.

### Bioinformatics analyses

Fragments were mapped to the tomato genome (ITAG3.2; from the International Tomato Genome Sequencing Project) using HiSat2 [65]. Counts per gene were generated using HTSeq [66] and the corresponding GTF file. Differential expression analysis of RNAseq data was conducted using negative binomial generalized linear models with DESeq2 [67]. We selected DESeq2 for analysis of our data because it has been reported to be robust, i.e. to exhibit a low false positive rate, for datasets with low number of replicates, as in our case [68].

DESeq2 is a moderate statistical method that compares gene counts in two groups by applying a modified Fisher’s exact test with at least 2 degrees of freedom. All our experiments had three degrees of freedom (n-1). Plants that were mock-inoculated served as reference for pairwise comparisons against plants inoculated either with *Fo*-npt or *Fol*-pt. Gene abundance differences with a corrected *p*-value < 0.05 were considered differentially expressed. No threshold for fold-change was established, because all values were over 1.58. Gene ontology analysis of differentially expressed genes was conducted with Blast2GO [33], with default parameters. Gene ontology terms with a corrected *p*-value < 0.05 were considered significantly enriched. Plots were generated with in-house R scripts. Additionally the PCA was conducted on Euclidean distances between samples derived from gene abundance (counts) for all transcripts detected following a regularized logarithmic transformation.

## Supplementary Information

**Additional file 1: Supplementary Figure 1.** PCA of control samples. Abundance (counts) of all detected genes, upon regularized logarithmic transformation, was used to calculate the Euclidean distances between mock samples of each cultivar. Such distances were used for PCA analysis and plotting.

**Additional file 2: Supplementary Fig. 2.** Intersection plot of differentially expressed genes. The number of genes that were commonly and differentially expressed between all pairs of comparisons are presented. Comparisons refer to control plants against plants inoculated with either *Fo*-npt or *Fol*-pt. **A**) Upregulated genes. **B**) Downregulated genes.

**Additional file 3: Supplementary Table S1.** Differentially expressed genes in the cultivar CT1 inoculated with Fo-npt. **Supplementary Table S2.** Differentially expressed genes in the cultivar CT1 inoculated with Fol-pt. **Supplementary Table S3.** Differentially expressed genes in the cultivar CT2 inoculated with Fo-npt. **Supplementary Table S4.** Differentially expressed genes in the cultivar CT2 inoculated with Fol-pt. **Supplementary Table S5.** Differentially expressed genes in the cultivar IAC391 inoculated with Fo-npt. **Supplementary Table S6.** Differentially expressed genes in the cultivar IAC391 inoculated with Fol-pt. **Supplementary Table S7.** Gene ontology analysis with BLAST2GO of genes found upregulated in cultivar CT1 inoculated with Fol-pt. **Supplementary Table S8.** Gene ontology analysis with BLAST2GO of genes found downregulated in cultivar CT2 inoculated with Fol-pt. **Supplementary Table S9.** Gene ontology analysis with BLAST2GO of genes found upregulated in cultivar IAC391 inoculated with Fol-pt. **Supplementary Table S10.** Gene ontology analysis with BLAST2GO of genes found downregulated in cultivar IAC391 inoculated with Fol-pt. **Supplementary Table S11.** Differentially expressed genes in the three cultivars per chromosome.

## Data Availability

Data generated is publicly available at the SRA portal of NCBI under accession number PRJNA606052.

## Abbreviations

*Fol*: *Fusarium oxysporum* f. sp. *lycopersici*
*Fol*-pt: *Fusarium oxysporum* f. sp. *lycopersici* pathogenic
*Fo*-npt: *Fusarium oxysporum* f. sp. *lycopersici* non*-*pathogenic
CT1: Commercial tomato cultivar 1
CT2: Commercial tomato cultivar 2
IAC391: Tomato cultivar 3
WRKY: Transcription factor
RNAseq: RNA sequencing
PAMPs: Pathogen-Associated Molecular Patterns
PRRs: Pattern Recognition Receptors
PTI: Pathogen-Triggered Immunity
ETS: Effector-Triggered Susceptibility
ETI: Effector-Triggered Immunity
PR: Pathogenesis-related proteins
(mi)-RNAs: Micro RNAs
hpi: Hours post-inoculation
PCA: Principal Component Analysis
CYP: Cytochrome P450
GO: Gene Ontology Analysis
ROS: Reactive Oxygen Species
RALF: Rapid Alkalinization Factor

## References

[1] McHale L, Tan X, Koehl P, Michelmore RW. Plant NBS-LRR proteins: adaptable guards. Genome Biol. 2006;7. 10.1186/gb-2006-7-4-212. 10.1186/gb-2006-7-4-212 PMC1557992 16677430

[2] Ma L-J, Geiser DM, Proctor RH, Rooney AP, O’Donnell K, Trail F (2013). *Fusarium* Pathogenomics. Annu Rev Microbiol.

[3] Jones JDG, Dangl JL (2006). The plant immune system. Nature.

[4] Heil M, Ton J (2008). Long-distance signalling in plant defence. Trends Plant Sci.

[5] Sun T, Zhang Y (2021). Short- and long-distance signaling in plant defense. Plant J.

[6] Edel-Hermann V, Lecomte C (2019). Current status of *fusarium oxysporum* Formae Speciales and races. Phytopathology..

[7] Snyder WC, Hansen HN (1940). The species concept in *Fusarium*. Am J Bot.

[8] Gordon TR (2017). *Fusarium oxysporum* and the *fusarium* wilt syndrome. Annu Rev Phytopathol.

[9] Enespa DSK (2014). Effectiveness of some antagonistic fungi and botanicals against *Fusarium* solani and *Fusarium oxysporum* f. sp. *lycopersici* infecting Brinjal and tomato plants. Asian J Plant Pathol.

[10] Inami K, Yoshioka-Akiyama C, Morita Y, Yamasaki M, Teraoka T, Arie T. A genetic mechanism for emergence of races in *Fusarium oxysporum* f. sp. *lycopersici*: inactivation of avirulence gene AVR1 by transposon insertion. PLoS One. 2012;7. 10.1371/journal.pone.0044101. 10.1371/journal.pone.0044101 PMC3428301 22952887

[11] Ortiz E, Cruz M, Melgarejo LM, Marquínez X, Hoyos-Carvajal L (2014). Características hispatologicas da infecção causada por *Fusarium oxysporum* e *F. solani* em maracujá-roxo (Passiflora edulis Sims). Summa Phytopathologica. Summa Phytopathol.

[12] Ignjatov M, Milosevic D, Nikolic Z, Gvozdanovic-Varga J, Jovicic D, Zdjelar G (2012). *Fusarium oxysporum* as causal agent of tomato wilt and fruit rot. Pesticidi i fitomedicina.

[13] da Silva JC, Bettiol W (2005). Potential of non-pathogenic *fusarium oxysporum* isolates for control of *fusarium* wilt of tomato. Fitopatol Bras.

[14] Validov SZ, Kamilova FD, Lugtenberg BJJ (2011). Monitoring of pathogenic and non-pathogenic *fusarium oxysporum* strains during tomato plant infection. Microb Biotechnol.

[15] Steinberg C, Lecomte C, Alabouvette C. Root interactions with nonpathogenic *fusarium oxysporum*: hey *fusarium oxysporum*, what do you do in life when you do not infect a plant? In: belowground defense strategies in plants. Sign Commun Plants. 2016. 10.1007/978-3-319-42319-7_12.

[16] Rep M, Van Der Does HC, Meijer M, Van Wijk R, Houterman PM, Dekker HL (2004). A small, cysteine-rich protein secreted by *fusarium oxysporum* during colonization of xylem vessels is required for I-3-mediated resistance in tomato. Mol Microbiol.

[17] Gonzalez-Cendales Y, Catanzariti AM, Baker B, Mcgrath DJ, Jones DA (2016). Identification of I-7 expands the repertoire of genes for resistance to *fusarium* wilt in tomato to three resistance gene classes. Mol Plant Pathol.

[18] Catanzariti A, Lim GTT (2015). Jones D a. the tomato I-3 gene : a novel gene for resistance to fusarium wilt disease. New Phytol.

[19] Takken F, Rep M (2010). The arms race between tomato and *fusarium oxysporum*. Mol Plant Pathol.

[20] Mandal S, Mallick N, Mitra A (2009). Salicylic acid-induced resistance to *fusarium oxysporum* f. sp. *lycopersici* in tomato. Plant Physiol Biochem.

[21] McGovern RJ (2015). Management of tomato diseases caused by *Fusarium oxysporum*. Crop Prot.

[22] Zhu QH, Stephen S, Kazan K, Jin G, Fan L, Taylor J (2013). Characterization of the defense transcriptome responsive to *Fusarium oxysporum*-infection in Arabidopsis using RNA-seq. Gene.

[23] Galindo-González L, Deyholos MK. RNA-seq transcriptome response of flax (*Linum usitatissimum* L.) to the pathogenic fungus *Fusarium oxysporum* f. sp. *lini*. Front Plant Sci. 2016;7. 10.3389/fpls.2016.01766. 10.3389/fpls.2016.01766 PMC5121121 27933082

[24] Thatcher LF, Williams AH, Garg G, Buck S-AG, Singh KB (2016). Transcriptome analysis of the fungal pathogen *Fusarium oxysporum* f sp *medicaginis* during colonisation of resistant and susceptible *Medicago truncatula* hosts identifies differential pathogenicity profiles and novel candidate effectors. BMC Genomics.

[25] Xing M, Lv H, Ma J, Xu D, Li H, Yang L, Kang J, Wang X, Fang Z (2016). Transcriptome profiling of resistance to *fusarium oxysporum* f. sp. *conglutinans* in cabbage (*Brassica oleracea*) roots. PLoS One.

[26] Ouyang S, Park G, Atamian HS, Han CS, Stajich JE, Kaloshian I (2014). MicroRNAs suppress NB domain genes in tomato that confer resistance to *fusarium oxysporum*. PLoS Pathog.

[27] Manzo D, Ferriello F, Puopolo G, Zoina A, D’Esposito D, Tardella L (2016). *Fusarium oxysporum* f.sp. *radicis-lycopersic*i induces distinct transcriptome reprogramming in resistant and susceptible isogenic tomato lines. BMC Plant Biol.

[28] Karasov TL, Chae E, Herman JJ, Bergelson J (2017). Mechanisms to mitigate the trade-off between growth and defense. Plant Cell.

[29] Phukan UJ, Jeena GS, Shukla RK (2016). WRKY transcription factors: molecular regulation and stress responses in plants. Front Plant Sci.

[30] Chen X, Li C, Wang H, Guo Z (2019). WRKY transcription factors: evolution, binding, and action. Phytopathol Res.

[31] Xu J, Wang XY, Guo WZ (2015). The cytochrome P450 superfamily: key players in plant development and defense. J Integr Agric.

[32] Saito A, Ooya T, Miyatsuchi D, Fuchigami H, Terakado K, Nakayama S-Y (2009). Molecular characterization and antifungal activity of a family 46 chitosanase from Amycolatopsis sp. CsO-2. FEMS Microbiol Lett.

[33] Conesa A, Gotz S, Garcia-Gomez JM, Terol J, Talon M, Robles M (2005). Blast2GO: a universal tool for annotation, visualization and analysis in functional genomics research. Bioinformatics..

[34] Zhao M, Ji H-M, Gao Y, Cao X-X, Mao H-Y, Liu P, et al. Comparative transcriptome profiling conferring of resistance to *Fusarium oxysporum* infection between resistant and susceptible tomato. bioRxiv. 2017:116988. 10.1101/116988.

[35] Zhao M, Ji H-M, Gao Y, Cao X-X, Mao H-Y, Liu P, et al. Integrated RNA-seq and sRNA-seq revealed differences in transcriptome between susceptible and resistant tomato responding to *Fusarium oxysporum*. bioRxiv. 2018:324574. 10.1101/324574.

[36] Bartholomew ES, Black K, Feng Z, Liu W, Shan N, Zhang X, et al. Comprehensive analysis of the chitinase gene family in cucumber (Cucumis sativus L.): from gene identification and evolution to expression in response to *fusarium oxysporum*. Int J Mol Sci. 2019;20(21). 10.3390/ijms20215309. 10.3390/ijms20215309 PMC6861899 31731414

[37] Dzhavakhiya VG, Ozeretskovskaya OL, Zinovyeva S V. Immune response. In: Comprehensive and molecular phytopathology. Elsevier; 2007. p. 265–314.

[38] Zhang J, Wang F, Liang F, Zhang Y, Ma L, Wang H (2018). Functional analysis of a pathogenesis-related thaumatin-like protein gene TaLr35PR5 from wheat induced by leaf rust fungus. BMC Plant Biol.

[39] Treutter D (2005). Significance of flavonoids in plant resistance and enhancement of their biosynthesis. Plant Biol.

[40] De Bruyne L, Höfte M, De Vleesschauwer D (2014). Connecting growth and defense: the emerging roles of brassinosteroids and gibberellins in plant innate immunity. Mol Plant.

[41] Upasani ML, Limaye BM, Gurjar GS, Kasibhatla SM, Joshi RR, Kadoo NY (2017). Chickpea-*fusarium oxysporum* interaction transcriptome reveals differential modulation of plant defense strategies. Sci Rep.

[42] Silvia Sebastiani M, Bagnaresi P, Sestili S, Biselli C, Zechini A, Orrù L, et al. Transcriptome analysis of the melon-*fusarium oxysporum* f. sp. *melonis* race 1.2 Pathosystem in susceptible and resistant plants. Front. Plant Sci. 2017;8. 10.3389/fpls.2017.00362. 10.3389/fpls.2017.00362 PMC5356040 28367157

[43] Singh B, Sharma RA (2015). Plant terpenes: defense responses, phylogenetic analysis, regulation and clinical applications. 3. Biotech..

[44] Kumari S, Priya P, Misra G, Yadav G (2013). Structural and biochemical perspectives in plant isoprenoid biosynthesis.

[45] Toffolatti SL, Maddalena G, Passera A, Casati P, Bianco PA, Quaglino F (2021). Role of terpenes in plant defense to biotic stress. Biocontrol agents and secondary metabolites.

[46] Iqbal Z, Iqbal MS, Hashem A, Abd Allah EF, Ansari MI (2021). Plant defense responses to biotic stress and its interplay with fluctuating dark/light conditions. Front Plant Sci.

[47] Trognitz F, Manosalva P, Gysin R, Niño-Liu D, Simon R, Herrera MDR (2002). Plant defense genes associated with quantitative resistance to potato late blight in *Solanum phureja* x dihaploid *S. tuberosum* hybrids. Mol Plant-Microbe Interact.

[48] Ishihara A, Hashimoto Y, Tanaka C, Dubouzet JG, Nakao T, Matsuda F (2008). The tryptophan pathway is involved in the defense responses of rice against pathogenic infection via serotonin production. Plant J.

[49] Iven T, König S, Singh S, Braus-Stromeyer SA, Bischoff M, Tietze LF (2012). Transcriptional activation and production of tryptophan-derived secondary metabolites in arabidopsis roots contributes to the defense against the fungal vascular pathogen verticillium longisporum. Mol Plant.

[50] Hiruma K, Fukunaga S, Bednarek P, Piślewska-Bednarek M, Watanabe S, Narusaka Y (2013). Glutathione and tryptophan metabolism are required for Arabidopsis immunity during the hypersensitive response to hemibiotrophs. Proc Natl Acad Sci U S A.

[51] Chen YC, Wong CL, Muzzi F, Vlaardingerbroek I, Kidd BN, Schenk PM. Root defense analysis against *fusarium oxysporum* reveals new regulators to confer resistance. Sci Rep. 2014;4(1). 10.1038/srep05584. 10.1038/srep05584 PMC4083284 24998294

[52] Zhang L, Du L, Poovaiah BW (2014). Calcium signaling and biotic defense responses in plants. Plant Signal Behav.

[53] Zhang H, Li A, Zhang Z, Huang Z, Lu P, Zhang D, et al. Ethylene response factor TERF1, regulated by ETHYLENE-INSENSITIVE3-like factors, functions in reactive oxygen species (ROS) scavenging in tobacco (*Nicotiana tabacum* L.). Sci Rep. 2016;6. 10.1038/srep29948. 10.1038/srep29948 PMC4951782 27435661

[54] Müller M, Munné-Bosch S (2015). Ethylene response factors: a key regulatory hub in hormone and stress signaling. Plant Physiol.

[55] Balasubramanian V, Vashisht D, Cletus J, Sakthivel N (2012). Plant β-1,3-glucanases: their biological functions and transgenic expression against phytopathogenic fungi. Biotechnol Lett.

[56] Segura A, Moreno M, Madueño F, Molina A, García-Olmedo F (1999). Snakin-1, a peptide from potato that is active against plant pathogens. Mol Plant-Microbe Interact.

[57] Berrocal-Lobo M, Segura A, Moreno M, López G, García-Olmedo F, Molina A (2002). Snakin-2, an antimicrobial peptide from potato whose gene is locally induced by wounding and responds to pathogen infection. Plant Physiol.

[58] Nahirñak V, Rivarola M, Almasia NI, Barón MPB, Hopp HE, Vile D, et al. Snakin-1 affects reactive oxygen species and ascorbic acid levels and hormone balance in potato. PLoS One. 2019;14. 10.1371/journal.pone.0214165. 10.1371/journal.pone.0214165 PMC6433472 30909287

[59] Herbel V, Sieber-Frank J, Wink M (2017). The antimicrobial peptide snakin-2 is upregulated in the defense response of tomatoes (*Solanum lycopersicum*) as part of the jasmonate-dependent signaling pathway. J Plant Physiol.

[60] Gupta S, Chakraborti D, Sengupta A, Basu D, Das S (2010). Primary metabolism of chickpea is the initial target of wound inducing early sensed *Fusarium oxysporum* f sp *ciceri* race I. PLoS One.

[61] Masachis S, Segorbe D, Turrà D, Leon-Ruiz M, Fürst U, El Ghalid M (2016). A fungal pathogen secretes plant alkalinizing peptides to increase infection. Nat Microbiol.

[62] Mizuno H, Katagiri S, Kanamori H, Mukai Y, Sasaki T, Matsumoto T (2020). Evolutionary dynamics and impacts of chromosome regions carrying R-gene clusters in rice. Sci Rep.

[63] Kankanala P, Nandety RS, Mysore KS (2019). Genomics of plant disease resistance in legumes. Front Plant Sci.

[64] Carmona SL, Burbano-David D, Gómez MR, Lopez W, Ceballos N, Castaño-Zapata J (2020). Characterization of pathogenic and nonpathogenic fusarium oxysporum isolates associated with commercial tomato crops in the Andean region of Colombia. Pathogens..

[65] Kim D, Langmead B, Salzberg SL (2015). HISAT: a fast spliced aligner with low memory requirements. Nat Methods.

[66] Anders S, Pyl PT, Huber W (2015). HTSeq--a python framework to work with high-throughput sequencing data. Bioinformatics..

[67] Love MI, Huber W, Anders S (2014). Moderated estimation of fold change and dispersion for RNA-seq data with DESeq2. Genome Biol.

[68] Schurch NJ, Schofield P, Gierliński M, Cole C, Sherstnev A, Singh V (2016). How many biological replicates are needed in an RNA-seq experiment and which differential expression tool should you use?. RNA..

