# Supplementary figures and images for: Transcriptional responses to Fusarium oxysporum f. sp. lycopersici (Sacc.) Snyder & Hansen infection in three Colombian tomato cultivars
Source: BMC Plant Biol. 2021 Sep 8;21:412. doi: 10.1186/s12870-021-03187-z (PMC8425103; doi:10.1186/s12870-021-03187-z)

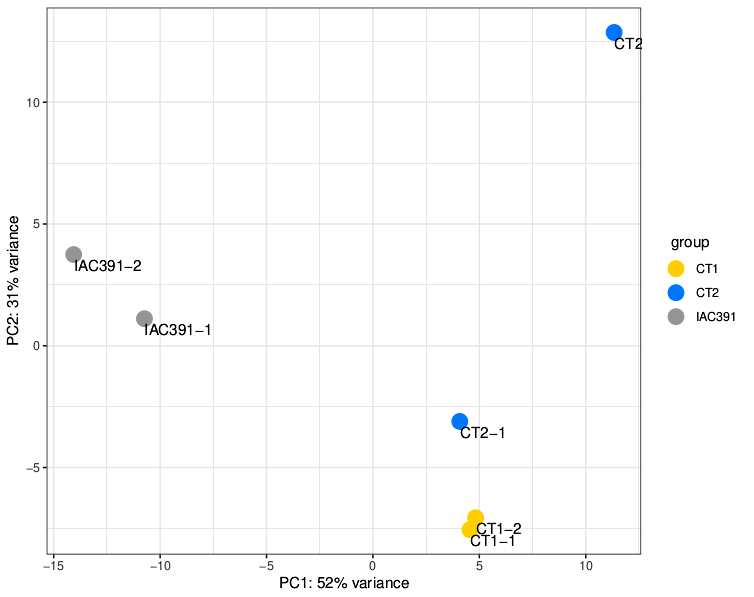

Supplement: Supplementary file 1 — Additional file 1: Supplementary Figure 1. PCA of control samples. Abundance (counts) of all detected genes, upon regularized logarithmic transformation, was used to calculate the Euclidean distances between mock samples of each cultivar. Such distances were used for PCA analysis and plotting. [file 12870_2021_3187_MOESM1_ESM.png]

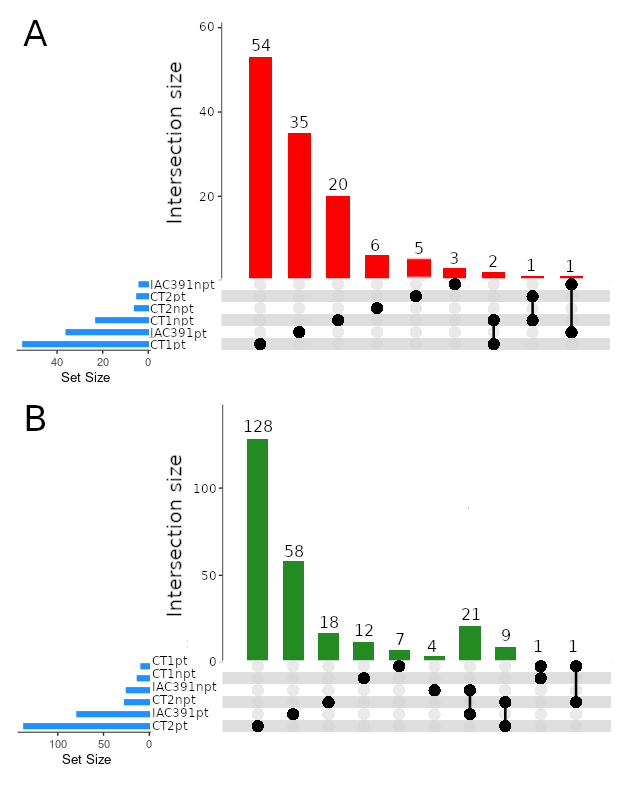

Supplement: Supplementary file 2 — Additional file 2: Supplementary Fig. 2. Intersection plot of differentially expressed genes. The number of genes that were commonly and differentially expressed between all pairs of comparisons are presented. Comparisons refer to control plants against plants inoculated with either Fo-npt or Fol-pt. A) Upregulated genes. B) Downregulated genes. [file 12870_2021_3187_MOESM2_ESM.png]
